# Does senescence promote fitness in *Caenorhabditis elegans* by causing death?

**DOI:** 10.1016/j.arr.2019.01.008

**Published:** 2019-03

**Authors:** Jennifer N. Lohr, Evgeniy R. Galimov, David Gems

**Affiliations:** Institute of Healthy Ageing, and Research Department of Genetics, Evolution and Environment, University College London, London WC1E 6BT, UK

**Keywords:** Adaptive death, Aging, Altruism, *C. elegans*, Evolution, Inclusive fitness

## Abstract

A widely appreciated conclusion from evolutionary theory is that senescence (aging) is of no adaptive value to the individual that it afflicts. Yet studies of *Caenorhabditis elegans* and *Saccharomyces cerevisiae* are increasingly revealing the presence of processes which actively cause senescence and death, leading some biogerontologists to wonder about the established theory. Here we argue that programmed death that increases fitness could occur in *C. elegans* and *S. cerevisiae*, and that this is consistent with the classic evolutionary theory of aging. This is because of the special conditions under which these organisms have evolved, particularly the existence of clonal populations with limited dispersal and, in the case of *C. elegans*, the brevity of the reproductive period caused by protandrous hermaphroditism. Under these conditions, death-promoting mechanisms could promote worm fitness by enhancing inclusive fitness, or worm colony fitness through group selection. Such altruistic, adaptive death is not expected to evolve in organisms with outbred, dispersed populations (e.g. most vertebrate species). The plausibility of adaptive death in *C. elegans* is supported by computer modelling studies, and new knowledge about the ecology of this species. To support these arguments we also review the biology of adaptive death, and distinguish three forms: consumer sacrifice, biomass sacrifice and defensive sacrifice.

## Highlights

• Senescent death of older inviduals could benefit younger kin.
• Theory rules out such programmed death in outbred, dispersed populations.
• *C. elegans* breed as non-dispersed, clonal populations of protandrous hermaphrodites.
• Under these conditions programmed, adaptive death could evolve.

## 1 Strained relations: *C. elegans* and the evolutionary theory of aging

Biogerontology, the study of the biology of aging, is a young field. Symptomatic of this is the continuing turnover of central concepts about aging. Here one example is the theory that reactive oxygen species (ROS) are a major cause of aging, and the idea that enhanced antioxidant defense should retard aging, now largely abandoned (Perez et al., 2009; Stuart et al., 2014; Van Raamsdonk and Hekimi, 2010). Another is the idea that retardation of aging by caloric restriction (CR) is a general feature of animals; but effects of CR on mouse lifespan have been shown to be strain dependent (Ingram and de Cabo, 2017; Liao et al., 2010; Mulvey et al., 2014) and results of CR studies on rhesus monkeys (Colman et al., 2009; Mattison et al., 2012) suggest that CR is unlikely to cause major retardation of aging humans in the way that it can do in rodents.

As these central pillars of biogerontology erode and crumble, those remaining become more important to support the field. Critical among these is the evolutionary theory of aging, which provides a powerful explanatory framework for understanding the origins of aging, the natural diversity in aging rate, and the value of aging as an adaptation - or rather, the lack of it (Rose, 1991). An important conclusion from evolutionary theory is that, as a rule, senescence serves no purpose, i.e. it does not contribute to biological fitness (the capacity of an organism to spread its genes in a population). This means that there should not be genes whose naturally selected function is to cause aging.

In this context, the discovery of single gene mutations that dramatically extend lifespan in *C.* elegans, e.g. those affecting the insulin/IGF-1 signaling (IIS) pathway (Kenyon, 2010), was unexpected. It also caused some discontent with evolutionary biology among some model organism biogerontogists. This has led to a few direct challenges to the classic evolutionary theory (Goldsmith, 2008; Longo et al., 2005; Mitteldorf, 2006; Skulachev, 2002), but more often it manifests in others ways, e.g. as hints that aging is programmed (Budovskaya et al., 2008), and slightly disdainful remarks about the theory, articulated in reviews (Kenyon, 2011) or to interviewers; for example, "[Evolutionary biologists] are always telling you what you can’t think" (Gary Ruvkun) (Anton, 2013).

What is going on here? Is it merely that some model organism geneticists lack understanding of the evolutionary theory? Is this just naive group selectionism on their part? Or is this a case of: there is no smoke without fire? I.e. are there substantive problems in terms of reconciling findings from model organism biogerontology with evolutionary theory? In this essay, we explore the nature of this inter-disciplinary tension, and present a diagnosis of the problem and a possible solution to it. We argue that there is indeed fire, but that the apparent discrepancy with classic evolutionary theory may be resolved by realizing that particular features of C. elegans (e.g. clonality, limited dispersal, protandrous hermaphroditism) are permissive for the natural selection of adaptive death (Dytham and Travis, 2006), i.e. programmed organismal death. Here death can be advantageous due to benefits in terms of inclusive fitness or colony-level fitness. In organisms where adaptive death occurs, promoting senescence and death can be a selected function of genes. In principle, the *daf-2* insulin/IGF-1 receptor gene in *C. elegans* could be such a gene, deserving its nickname of "the grim reaper" (Anton, 2013).

## 2 The beautiful evolutionary theory of aging

Biogerontologists who dislike the evolutionary theory of aging are missing out, since it is a thing of great explanatory power and beauty. It argues that aging is not itself adaptive, but rather a non-selected evolutionary by-product caused by a decline in natural selection with increasing age (Charlesworth, 1993, 2000, 2001; Hamilton, 1966). Two theories have been proposed for how aging evolves: mutation accumulation (MA) and antagonistic pleiotropy (AP). According to the MA theory, inheritable, late-acting deleterious mutations accumulate in populations, leading to late-life disease and an age increase in death rate (Medawar, 1952). For example, a mutant allele that kills young children will be strongly selected against (as it will not be passed to the next generation), while a lethal mutation with effects confined to people over the age of 80 will experience little selection (people with this mutation will have already passed it to their offspring by that age). The AP theory takes into account the fact that gene mutations can be pleiotropic, i.e. have multiple effects at different places and different times. Where a new allele enhances early-life fitness but promotes disease in later life, it may enhance fitness overall due to the greater impact on fitness of the early effect, in which case frequency of the new allele is likely to increase due to natural selection (Williams, 1957). Thus, although the AP gene variant increases fitness, its promotion of senescence does not (Bourke, 2007).

In terms of the biological mechanisms through which it acts, AP may be viewed in several different ways, as follows. First, as a property of individual structural genes, as in G.C. Williams’ hypothetical example of a gene promoting calcium deposition that in early life enhances bone growth and thereby fitness (e.g. through capacity to run away from predators), but in later life promotes calcium deposition in arteries, leading to cardiovascular disease (Williams, 1957). Next, AP may be viewed as a property of regulatory genes controlling entire programs of growth and differentiation, promoting fitness in early life, but running on in later life in a futile fashion to become pathogenic quasi-programs or pseudo-programs (Blagosklonny, 2013, 2006; de Magalhães, 2012); wild-type *daf-2* in *C. elegans* is a potential example this form of AP (Blagosklonny, 2010; Ezcurra et al., 2018). Third, AP may be viewed as a consequence of biological constraints resulting e.g. from developmental or architectural features of organisms (or *Baupläne*) (Gould and Lewontin, 1979).

As an illustration of the latter, consider the evolution by natural selection of hermaphroditism in C. elegans from the ancestral gonochoristic state (i.e. females and males) (Ellis and Lin, 2014; Kiontke et al., 2011). The germline of C. elegans hermaphrodites is protandrous, i.e. forming sperm first (∼300) and then oocytes. Sperm are stored in sacs, the spermathecae, through which oocytes pass and are fertilized. Hermaphroditism is thought to have been selected for because it promotes fitness by accelerating population growth rate, allowing *C. elegans* to outcompete rivals for colonization of transient food sources, typically rotting plant stems and fruit (Schulenburg and Félix, 2017). But as a *Bauplan* protandry is subject to a major constraint: the switch from sperm to oocyte production initiates egg laying, but it also limits sperm number; once the sperm are depleted, *C. elegans* cannot reinitiate sperm production. Interestingly, the weakly masculinizing mutation *tra-3(e2333)* delays the switch from sperm to egg production, thereby markedly increasing brood size which, one might reason, ought to increase fitness. In fact, *tra-3(e2333)* reduces population growth rate, because the additional time required to make the extra sperm causes a delay in the onset of egg laying (Hodgkin and Barnes, 1991). Thus, *tra-3(e2333)* exhibits *Bauplan*-type AP, reducing early life fitness (by delaying the onset of egg laying) but increasing later fitness (by increasing brood size).

Overall, the evolutionary theory of aging provides an explanation for why aging exists and how it evolves. It predicts that aging serves no biological function but is a mere evolutionary epiphenomenon. Aging is pointless and harmful. Consequently, the notion that aging might be a positively selected death program has in the past been vehemently rejected by evolutionary biologists (Austad, 2004; Kirkwood, 2005).

## 3 Features of *C. elegans* and *S. cerevisiae* aging suggest programmed aging

The rigor and explanatory power of the evolutionary theory notwithstanding, some features of aging in certain model organisms seem to suggest some form of adaptive benefit from aging. These include (i) the occurrence of programmed cell death in *Saccharomyces cerevisiae*, a unicellular organism (Gourlay et al., 2006); (ii) the fact that large increases in lifespan can occur without reductions in fertility (Kenyon, 2005); (iii) the existence of genes which when mutated cause large increases in *C. elegans* lifespan, particularly genes in the IIS pathway such *daf-2* and *age-1* (phosphatidylinositol-3 kinase), mutation of which can increase worm lifespan as much as 10-fold (Ayyadevara et al., 2008); and (iv) the occurrence of destructive processes promoting senescence within days or even hours of reproductive maturity and with no apparent linked fitness benefit (Labbadia and Morimoto, 2014).

### 3.1 Defining programmed aging

To explore these issues further it is helpful to clarify the terminology employed. The term *programmed aging* is somewhat confusing, such that it has even been argued that its use should be discontinued (Bourke, 2007; Rose, 1991). However, we believe that with a little disambiguation and adjustment, the term can be made unambiguous and useful. Its first problem relates to the word *aging*, which has multiple meanings that are sometimes conflated (Janac et al., 2017). These include benign developmental changes occurring during adulthood (*maturation*), and age changes that are wholly deteriorative (*senescence*). In recent years, this usage of *senescence* is sometimes confused with *cellular senescence*, a term introduced by Hayflick to describe a particular type of change that affects dividing mammalian cells *in vitro* (Hayflick and Moorhead, 1961). Cellular senescence is now understood to play a role in development and wound healing as well as organismal senescence (Campisi, 2013; Munoz-Espin et al., 2013). In this essay, when we say *aging*, we mean senescence in the original meaning of the word.

The concept of programmed senescence views aging as "caused by an ordered series of molecular events which are genetically coded in ways that may be similar to the ways developmental processes are coded" (Johnson and McCaffrey, 1985). Arguably, a shortcoming of this definition is that it is ambiguous as to whether or not *programmed* here implies a process that contributes to fitness, particularly since the word "programmed" implies purpose (Austad, 2004). To clarify this, the term *quasi-programmed* was introduced, to denote development-like processes that promote fitness in early life that run on in later life and promote senescence but not fitness (Blagosklonny, 2006); a good example of this in *C. elegans* is uterine tumour formation (Wang et al., 2018). To reduce ambiguity in this review, we refer to adaptive and non-adaptive programmed aging as programmed and quasi-programmed, respectively. By this terminology, and based on evolutionary theory, the default expectation is that aging in *C. elegans* may be quasi-programmed, but we are asking: could it actually be programmed? Various observations suggest the latter, and the broader occurrence of programmed aging in certain organisms, as follows.

### 3.2 Why do unicells exhibit programmed cell death (PCD)?

PCD mechanisms such as apoptosis contribute to fitness in metazoan organisms by various means but not, it is assumed, by promoting organismal death. This implies that in unicellular organisms, where PCD and programmed organismal death (POD) are one and the same, PCD should not exist. Yet in fact budding yeast (*S. cerevisiae*) do exhibit PCD (Fabrizio et al., 2004; Fabrizio and Longo, 2008; Gourlay et al., 2006). Indeed, the apparent occurrence of POD in budding yeast has been cited in challenges to the view that aging is non-adaptive (Longo et al., 2005; Skulachev, 2002).

### 3.3 Life extension without reduced fertility

One reason for irritation with the evolutionary theory among model organism biogerontologists is a sense that it led to incorrect conclusions that obstructed the initial development of lifespan genetics (Kenyon, 2011). There is surely some truth to this belief, at least insofar as the classic theory suggests that "genes for aging" should not exist.

The origins of *C. elegans* lifespan genetics lies with the work of Michael Klass who, in a brilliantly original study, first demonstrated that it is possible to isolate long-lived *C. elegans* mutants (Klass, 1983). However, Klass interpreted the longevity of the mutants as the result (in most cases) of feeding defects leading to dietary restriction, and concluded that "it is most probable that none of the mutants with increased life spans bear mutations in specific aging genes" (Klass, 1983). With hindsight, it seems strange that the crucial implications of these findings should have been ruled out on this way. Was this the malign influence of the evolutionary theory at work? According to Klass he was well aware of the evolutionary theory when he conducted the study. But his negative conclusions (which were present in the submitted manuscript) were based on his earlier work demonstrating that dietary restriction can increase lifespan in *C. elegans* (Klass, 1977), and the presence of feeding defects in most of his long-lived mutants (M.R. Klass, personal communication).

Characterising one of Klass's mutants his colleague Tom Johnson subsequently showed that life-extension was not due to dietary restriction. Instead it was shown in a seminal study that a defined mutation, *age-1(hx546)*, dramatically increases both healthspan and lifespan (Friedman and Johnson, 1988). However, again the authors were reluctant to conclude that wild-type *age-1* acts in some direct way to promote aging. Instead attention was drawn to an apparent reduction in *age-1* mutant fertility, and it was concluded that "It is likely that the action of *age-1* in lengthening life results not from eliminating a programmed aging function but rather from reduced hermaphrodite self-fertility or from some other unknown metabolic or physiologic alteration." It was subsequently shown that reducing IIS can increase lifespan without reducing fertility (Gems et al., 1998; Johnson et al., 1993). Moreover, preventing hermaphrodite reproduction altogether does not increase lifespan (Friedman and Johnson, 1988; Kenyon et al., 1993; Klass, 1977). Subsequently, dissociation of effects on lifespan and fertility have been demonstrated in a variety of contexts (Grandison et al., 2009; Kenyon, 2004, 2005; Piper et al., 2017).

### 3.4 Why does IIS shorten lifespan?

Mutation of *daf-2* can greatly increase *C. elegans* lifespan (Kenyon et al., 1993). This fact taken alone suggests that *daf-2* has evolved to cause death, which somehow promotes fitness. Because evolutionary theory argues against this, various attempts have been made to explain away this apparent contradiction to it. One proposal was that *daf-2* controls the switch between development to adulthood and dauer larva developmental arrest. Since dauer larvae are much longer lived that wild-type adults (Klass and Hirsh, 1976), it was suggested that the longevity of *daf-2* mutant adults is attributable to mis-expression of dauer longevity-assurance mechanisms in the adult (Kenyon et al., 1993), and comparisons of gene expression profiles of dauers and *daf-2* mutants supported this idea (McElwee et al., 2003, 2004). However, more recently it was discovered that *daf-2* mutant longevity can be dissociated from such dauer-ness (Ewald et al., 2015). Another suggested solution is that IIS is a conserved, nutrient-sensitive regulator of trade offs between reproduction and somatic maintenance (survival), enabling life history plasticity in the face of a changing nutrient environment (Partridge and Gems, 2002). Yet it appears increasingly doubtful that inadequate somatic maintenance (e.g. prevention and repair of stochastic molecular damage) is a major cause of aging in *C. elegans* (Ezcurra et al., 2018; Gems and Partridge, 2013). A third possibility, explored here, is that by promoting death *daf-2* somehow promotes fitness.

### 3.5 Why does *C. elegans* possess active mechanisms of self-destruction?

The last decade has seen discoveries that further strain the plausibility of the claim that *C. elegans* aging is non-adaptive. Most strikingly, it was discovered that mechanisms that protect protein folding homeostasis undergo a major collapse shortly after reproductive maturity. This collapse is detectable by day 3 of adulthood as a dramatic decline in heat stress resistance, and an ∼80% reduction in induction after stress challenge of genes involved in the heat shock and unfolded protein responses (Ben-Zvi et al., 2009; Shemesh et al., 2013; Taylor and Dillin, 2013), followed by increased levels of protein aggregation (Ben-Zvi et al., 2009; David et al., 2010). More recently, it was shown that this collapse occurs, startlingly, within 6 h of reproductive maturity, and is promoted by signaling from the germline (Labbadia and Morimoto, 2015, 2014). The reports on the proteostatic collapse phenomenon present no evidence of a coupled increase in fitness; but when interviewed a key senior author of these studies (R.I. Morimoto) remarked: "I absolutely believe that aging is programmed" (Anton, 2013).

Does this mean quasi-programmed or actually programmed? In principle, it could be either. One possibility is that proteostatic collapse is somehow coupled to individual fitness, e.g. by reducing the constraints imposed by protein quality control on protein synthesis rate (Sherman and Qian, 2013) or secretion (Labbadia and Morimoto, 2014) (i.e. quantity over quality), thereby increasing yolk production capacity - and fitness. Here the collapse process is quasi-programmed AP, i.e. proteostatic collapse does not in itself promote fitness but is merely an unselected by-product. To be precise, according to this scenario reducing protein folding capacity has two consequences: increasing yolk production (programmed) and proteostatic collapse (quasi-programmed). Another possibility is that promotion of death promotes fitness though inclusive fitness effects (detailed below); here the process is programmed and not AP, i.e. death itself actually promotes fitness, rather than being an unselected by-product of some other trait that does. Several authors of these studies view both possibilities as plausible (J. Labbadia and R.I. Morimoto, personal communication).

A further explanation that has been suggested to explain proteostatic collapse draws on the disposable soma theory. This theory argues that aging is caused by accumulation of stochastic molecular damage, and that fitness is maximised by partition of available resources (e.g. caloric) optimally between reproduction and somatic maintenance, in a manner sufficient only to assure longevity during the reproductive period (Kirkwood, 1977). Thus, down-regulating maintenance of proteostasis could increase resources available for reproduction at the cost of reduced protection of the soma, and reduced lifespan (Ben-Zvi et al., 2009; Labbadia and Morimoto, 2015, 2014; Shai et al., 2014). (Arguably, increasing fidelity of protein synthesis by slowing translation rate, as noted above, is not a somatic maintenance mechanism in this sense). However, it remains unclear that stochastic molecular damage is a major cause of senescence in *C. elegans*, and other proximate mechanisms have been identified (Ezcurra et al., 2018; Gems and Partridge, 2013; Van Raamsdonk and Hekimi, 2010).

Since the discovery of proteostatic collapse in 2009, more studies have appeared documenting other examples of early deterioration in *C. elegans*. For example, long-term memory is impaired by days 2–3 of adulthood and is entirely lost by day 5 (Kauffman et al., 2010), and degeneration of the dendrites of PVD neurons occurs from ∼day 4–7 that is actively promoted by the antimicrobial peptide NLP-29 and driven by autophagy (E et al., 2018). Also, in early adulthood *C. elegans* neurons extrude membrane-bound vesicles full of aggregated proteins and organelles (exophers), suggesting pathological upheaval at this time (Melentijevic et al., 2017). More broadly, analysis of the temporal dynamics of development of a range of senescent pathologies showed concerted disease development largely occurring from day 4–12 of adulthood (Ezcurra et al., 2018). These pathologies include intestinal atrophy and yolk steatosis, which are coupled in an IIS-promoted process of gut-to-yolk biomass conversion, suggesting quasi-programmed AP action in this case (Ezcurra et al., 2018). In addition, some features of organismal death suggest programmed or quasi-programmed function, such as waves of cellular necrosis promoted by calcium influx accompanying rigor mortis and intestinal death (Coburn et al., 2013; Galimov et al., 2018).

Altogether, one can see how these results might seem to constitute some kind of challenge to the classic evolutionary theory of aging, and certainly explain the ambivalent attitude to it among model organism biogerontologists. Although all these phenomena may eventually be explicable in terms of AP, they also suggest that it is worth considering more carefully the possibility that death might somehow promote fitness in *C. elegans* and *S. cerevisiae*.

## 4 Reconsidering the possibility of death as an adaptive trait

To explore this possibility, let us examine in more detail the concept of adaptive death. The idea that aging and death are beneficial has a long history (Gruman, 1966). The Roman philosopher Lucretius argued that death allows one generation to make way for the next (Bailey, 1963). Darwin's contemporaries August Weismann and Alfred Russel Wallace reasoned that aging evolved because it benefits the species by removing worn out individuals, and thereby reducing competition for limited resources (Rose, 1991). But at that time evolutionary theory did not clearly distinguish selection within and between species (Kirkwood and Cremer, 1982). With the appearance of modern evolutionary genetics, it was realized that selection between individuals in groups of organisms is far stronger than selection between groups, making altruistic death of this sort highly unlikely (Maynard Smith, 1976; Williams, 1966).

**Fig. 1:**
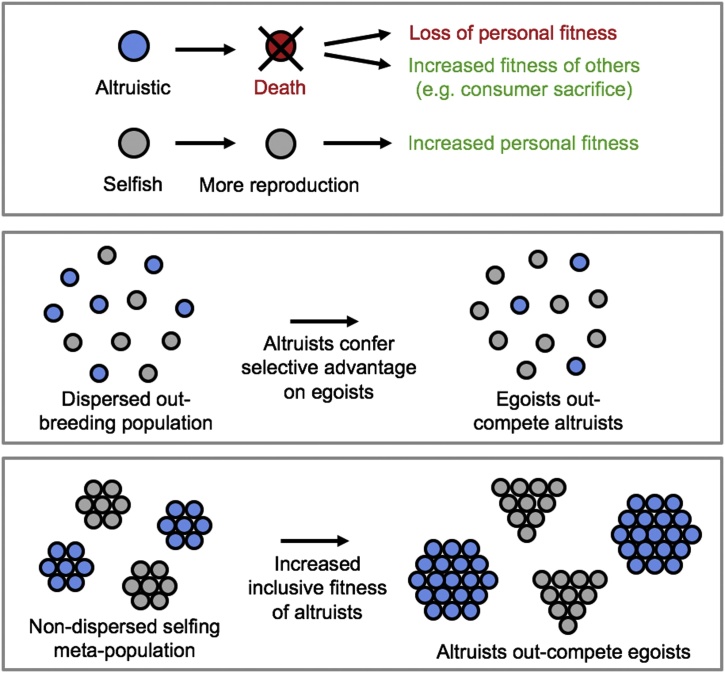
General conditions for the evolution of altruism by natural section. This scheme illustrates how altruism may either decrease or increase fitness, depending on conditions. Top, two variants exist in an organism, one altruistic (altruists, blue) and the other selfish (egoists, grey). Middle, in a dispersed, outbreeding population (e.g. fruitflies, most mammals), egoists exploit altruists, and therefore have greater fitness. Bottom, given viscous, clonal populations (e.g. colonial microbes, and perhaps *C. elegans*), altruists benefit from one another, and out-reproduce egoists, and therefore have greater fitness. Adaptive death is an altruistic behavior.

In principle, it might seem prudent for a population of animals to all age and die after a set time in order to conserve limited food supplies and avoid starvation. However, under normal circumstances a mutation generating a selfish cheat phenotype that refused to senesce but instead carried on reproducing is predicted to outcompete its altruistic brethren (Fig. 1), even though this might doom the entire population to starvation. The same principle is operational in human social contexts where the benefits of human altruism are undermined by selfish cheats, sometimes referred to as "the tragedy of the commons" (Rankin et al., 2007). Because the cost to altruistic aging individuals in terms of lost reproductive effort via reduced lifespan is predicted to exceed the benefit it provides to the group, this view of aging as adaptive is widely rejected (Rose, 1991; Williams, 1966).

### 4.1 Evolution of altruism by kin selection

While taking it as given that senescence is not usually adaptive, could it ever be? This is a question about the evolution of altruism (social behaviour entailing a net cost in lifetime offspring output to the actor). Here arguments largely entail two related theoretical frameworks, *inclusive fitness* and *multilevel selection* (including group selection)(Gardner et al., 2011; Lehtonen, 2016; Marshall, 2015; West et al., 2007). Natural selection favors genotypes that maximize fitness, and here two metrics may be distinguished. The first is the number of offspring that an individual itself begets, sometimes referred to as its *personal fitness* (or Darwinian fitness). The second is the number of offspring that an individual generates through its behavior (regardless of who actually begets them), its *inclusive fitness* (Hamilton, 1964). Thus, personal fitness is a subset of inclusive fitness. Social behavior, including altruism, can evolve via kin selection thanks to benefits to the individual in terms of inclusive fitness. For example, the altruistic behavior of honey bee workers has been selected for due to inclusive fitness benefits in the complete absence of personal fitness (since they are sterile).

Whether altruism can evolve for the benefit of all individuals within a group, or whether it will be spoiled by invasion of selfish cheats is condition dependent. This follows from Hamilton's rule *rB* > *C* where *r* is the relatedness, *B* is the benefit to the recipient, and *C* is the cost to the donor (Hamilton, 1964). A high value of *r* reduces the likelihood of cheat invasion; hence in clonal populations, for example, it is easier for altruism to evolve (Fig. 1). Evolution of altruism is also facilitated by low dispersal, i.e. such that related individuals remain in close proximity to one another, as opposed to dispersing and mixing with unrelated individuals; such populations are sometimes referred to as *viscous* (Trivers, 1971; Van Baalen and Rand, 1998).

### 4.2 Evolution of altruism by group selection

Evolution by natural selection largely occurs due to differential reproduction of individuals, but it can occur due to differential survival of groups, for example of honey bee colonies (Seeley, 1997). However, how one defines "individual" and "group" is to a certain extent a matter of perspective. Altruistic behavior of cells within clonal populations was key to the evolution of multicellular organisms, which from one perspective are complex, colonial, high viscosity societies of cells, exhibiting a high degree of altruism which promote their individual inclusive fitness (Bourke, 2011). In a similar fashion, in colonies of metazoan kin (e.g. honey bees) individuals show altruistic behavior that promotes their individual inclusive fitness. In each case the individual (cell, organism) increases its inclusive fitness by promoting the individual fitness of a higher order entity (organism, super-organism).

### 4.3 Multilevel selection (MLS)

This perspective reveals, beyond a simple individual vs group dichotomy, a hierarchy of different levels of organisation, which can be extended down to competition between genes within cells, and up to higher order groups. That natural selection is acting simultaneously at each level creates a complex situation that has been likened to Russian matryoshka dolls nested one inside the other (Wilson and Wilson, 2008). Analyzing this is the goal of MLS analysis.

MLS analysis is an updated form of group selection analysis, of which two forms may be distinguished, MLS-1 and MLS-2. MLS-1 considers how natural selection affects frequency of individuals within a group, and MLS-2 focuses on selection at the level of differential survival and reproduction of groups within a meta-population of groups (Kramer and Meunier, 2016). For example, MLS-2 analysis would be applicable to understanding differential survival of beehives, and establishment of descendent hives. The current, general consensus is that MLS-1 and inclusive fitness analysis are essentially equivalent frameworks, though not everyone agrees with this; for a detailed discussion see (Kramer and Meunier, 2016).

These developments in evolutionary biology have led to a renewed interest in the role of altruism in the evolution of aging, and the possibility that programmed death could, under some conditions, promote fitness (Bourke, 2007; Dytham and Travis, 2006; Lee, 2003; Longo et al., 2005; Markov, 2012; Mitteldorf, 2006; Travis, 2004). To explore this further with particular reference to *C. elegans*, we will first develop some additional concepts and terminology.

## 5 Mechanisms of adaptive death: consumer sacrifice and biomass sacrifice

Hypotheses about the fitness benefits of a given feature of an organism can be difficult to prove or disprove (Williams, 1966), and concerning possible benefits of senescence this is certainly true. Given that this essay presents hypotheses but does not prove them, we can at least be clear about what the hypotheses are - and what they are not.

**Fig. 2:**
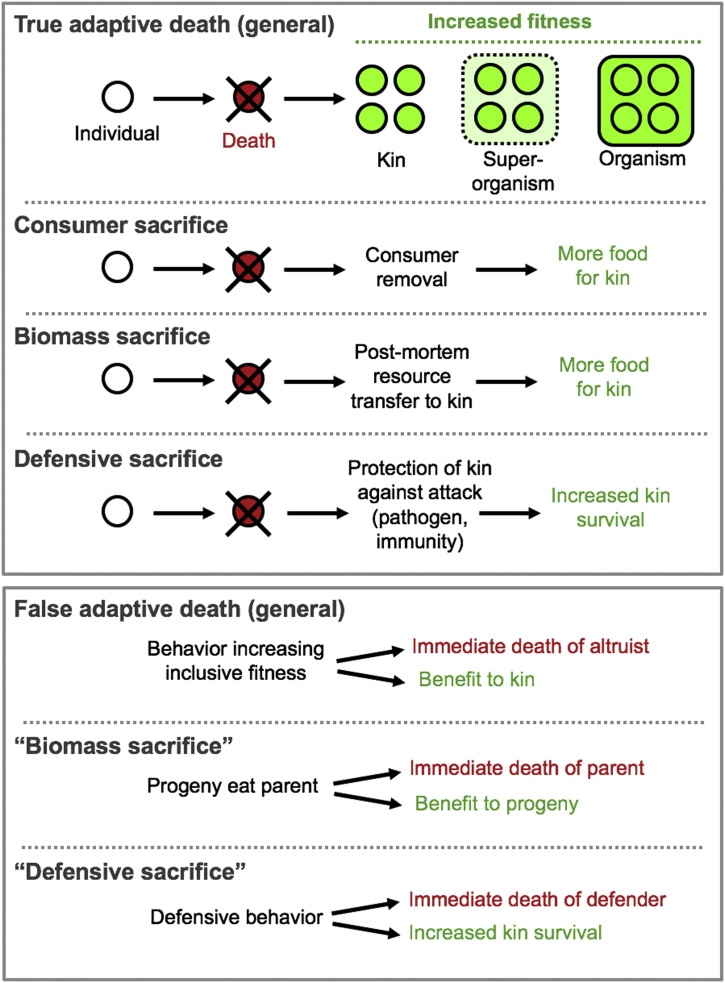
Defining adaptive death. Top panel, true adaptive death. Here death of an individual increases its fitness through effects on inclusive fitness and / or by increasing fitness of a super-organism (e.g. *S. cerevisiae* colony) or organism (e.g. cells within an organism). Three forms of adaptive death are defined. Adaptive death is typically envisaged as consumer sacrifice; here two possible additional forms of adaptive death are defined. As potential examples: biomass sacrifice, *S. cerevisiae* where programmed cell death may support growth elsewhere in the yeast colony through release of nutrients (Váchová et al., 2012); defensive sacrifice, *Leishmania* spp., where programmed cell death of protozoan parasites under immune defense may blunt the host immune response against kin (Zangger et al., 2002). Colors represent fitness changes; red, cost; green, gain. Bottom panel, false adaptive death. Here death is a consequence of behaviors that increase inclusive fitness, but is not in itself adaptive. The difference between false adaptive death and other forms of fitness *vs* lifespan trade offs is only that death occurs immediately as a consequence of fitness-promoting behavior. As potential examples: "biomass sacrifice", matriphagy in black lace-weaver spiders; "defensive sacrifice", hive defense by honey bees, where stinging behavior is fatal to the deliverer.

The main hypothesis here is that death *per se*, and the senescent pathologies that increase its probability, promote fitness - i.e. that species exist which exhibit adaptive death, or programmed organismal death (POD)(Fig. 2, top). This is quite different to the idea that senescence is coupled to fitness through pleiotropy or trade offs. As an example of what adaptive death is not, consider the honey bee, where a worker who stings a threatening human being may die as the result of deploying the sting. Here the act of stinging may be beneficial in fitness terms (by protecting the hive), but the resulting death of the bee is not (*false adaptive death*, Fig. 2, bottom). The difference between false adaptive death and other forms of fitness *vs* lifespan trade offs is only that death occurs immediately as a consequence of fitness-promoting behavior; here death *per se*, like senescence *per se*, provides no benefit.

### 5.1 Consumer sacrifice

In Weismann and Russel Wallace's account death itself promotes fitness by reducing competition for food and other resources. We here refer to this mechanism of adaptive death as *consumer sacrifice* (Fig. 2, top), since its benefits issues from elimination of a consumer. This mechanism requires strong kin or group selection in order to evolve.

### 5.2 Biomass sacrifice

A second mechanism of adaptive death may also be defined that involves a post-mortem trade off with enhanced reproductive fitness. In standard reproduction-aging trade offs, organisms can transfer resources to relatives and die as a consequence, and this can happen either soon after the transfer (immediate cost) or later (delayed cost). An additional possibility is that by dying a parent can enable or enhance resource transfer to progeny. Here all are forms of trade off, but the latter is a special case of a death-mediated resource transfer, that we will call *biomass sacrifice*. In this case, in contrast to most trade offs, the cost comes first and then the benefit, making death a selected phenotype. A key question relating to any potential instance of biomass sacrifice-type adaptive death is whether the death event itself could plausibly promote fitness.

A possible (but unproven) example of biomass sacrifice-type adaptive death for which there is some supporting evidence involves those species of Pacific salmon (*Oncorhynchus spp*.) which die rapidly after spawning. Here death occurs as a consequence of pathologies incurred due to physiological changes linked to reproductive effort, and the exertions of swimming large distances up river, and is an example of *reproductive death*, an extreme example of a trade off between reproduction and lifespan (Finch, 1990). However, an additional, and not mutually exclusive possibility is that by dying in the upper reaches of nutrient poor rivers and streams, salmon enable post-mortem resource transfer to their offspring or other kin, hatched from eggs laid in the same streams. Results from several studies suggest that nutrients released by decaying corpses of exhausted salmon can aid the growth and development of salmon fry (Bilby et al., 1998; Cederholm et al., 1999). In particular, nitrogen and phosphorus compounds released from carcasses support growth of phytoplankton and zooplankton upon which salmon fry feed (Cederholm et al., 1999). The possible existence of such adaptive death in semelparous salmon warrants further investigation.

### 5.3 Is matriphagy adaptive death?

Is death from the internal hatching of larvae (or *endotoky*) in *C. elegans* a biomass sacrifice-type adaptive death? This occurs when gravid hermaphrodites actively retain within the uterus fertilized eggs that they would otherwise lay. The result is that the larvae hatch internally, and devour their mother (matriphagy), leading eventually to a so-called *bag-of-worms* state (or *bag*), where larvae are trapped within the residual maternal cuticle. Such bagging occurs when food levels are low, and in the presence of pathogenic bacteria or toxins (Calafato et al., 2008; Chen and Caswell-Chen, 2003, 2004; Mosser et al., 2011; Van Raamsdonk and Hekimi, 2009). Consumption of maternal biomass by internally hatched larvae may increase their probability of survival, particularly under starvation conditions where it may enable larvae to reach the dauer larva dispersal stage (Chen and Caswell-Chen, 2003, 2004).

That larvae remain trapped inside a durable and non-permeable cuticle for some time after maternal death may be significant: even when the mother is dead, resources derived from her are protected inside her cuticle from exploitation by non-relatives (including other species). The post-mortem durability of the cuticle could, for this reason, be a selected trait. This scenario illustrates a more general point: that any conditions which assure receipt of resources released by parental death by their progeny can facilitate the evolution of adaptive death (biomass sacrifice or consumer sacrifice).

But is bagging really an example of adaptive death? Egg retention by gravid hermaphrodites is a regulated behavior. If bagging is an adaptation to increase progeny survival by biomass transfer, then the decision to deliberately withhold eggs and bag is also a decision to die. However, if the mother dies due to her progeny eating her, then death is a *consequence* of resource transfer, not a mediator of it. This is similar to the case of the honey bee's sting, i.e. death *per se* is not adaptive (false adaptive death, Fig. 2, bottom). Yet the mechanisms of organismal death during bagging have not been defined, and it remains possible that in addition to egg retention, mothers trigger other programmed mechanisms of organismal death to hasten resource transfer to their offspring. For example, during organismal death in *C. elegans*, a wave of necrotic cell death occurs in the intestine, which has been suggested to serve this function (Coburn et al., 2013).

This discussion may be usefully extended to other examples of matriphagy, as occurs for example in a number of species of insects and spiders, where it has been shown to increase fitness not only of offspring but also of mothers (Salomon et al., 2005; Suzuki et al., 2005). This is illustrated by the black lace-weaver spider (*Amaurobius ferox*) where mothers that are cannibalized have higher fitness than those that are not and go on to produce a second clutch (Kim et al., 2001). However, again, in most cases this appears to be reproductive death rather than adaptive death (Fig. 2, bottom), though *A. ferox* mothers clearly invite their offspring to eat them (Kim and Horel, 1998), and in the spider *Stegodyphus mimosarum* the mother liquefies her own internal organs which are then sucked out by her offspring, leading to her death (Seibt and Wickler, 1987).

These examples show that distinguishing biomass sacrifice-type adaptive death from reproductive death can be difficult. Yes, in principle one may distinguish two cases: (a) maternal death promoting resource transfer, and (b) resource transfer promoting maternal death. However, during matriphagy both may be happening at the same time and difficult to disentangle, particularly since organismal death is an extended process rather than an instantaneous event (Galimov et al., 2018). The onset of matriphagy (false adaptive death) may well activate mechanisms of death (e.g. necrosis) that hasten resource transfer (true adaptive death) (Fig. 2). Thus, to identify adaptive death in some cases, a more useful question than: "Is it (a) rather than (b)?" may be: "Does (a) promote fitness to any degree?"

Thus far we have established that some findings from model organisms appear strange in the light of the evolutionary theory of aging. We have prepared the ground to address this by disambiguating biogerontological terminology, re-examining arguments against adaptive aging, and introducing some new concepts and terminology. With this groundwork, in the next sections we will attempt to reconcile the strange findings with the theory, first considering the significance of programmed cell death in unicellular organisms, and then asking whether either biomass sacrifice or consumer sacrifice could plausibly occur in *C. elegans*.

## 6 Programmed cell death as programmed organismal death?

For multicellular organisms the benefits of PCD via apoptosis are many, including regulation of growth and development, protection against infection, and against cancer (Duncan et al., 2000; Evan, 1994; Reed, 1994). But for unicellular organisms, PCD is equivalent to POD. Thus, from the classic evolutionary theory of aging, one might expect that PCD would be absent from unicellular organisms. In fact, this is far from being the case. There are conserved genetic pathways that control apoptosis during PCD from prokaryotes to eukaryotes (Aravind et al., 1999; Gordeeva et al., 2004; Koonin and Aravind, 2002), suggesting that PCD evolved before the appearance of multicellularity.

In *S. cerevisiae*, the existence of apoptotic molecular machinery and occurrence of programmed cell death is well established (Carmona-Gutierrez et al., 2018; Gourlay et al., 2006; Hardwick, 2018), the evolutionary significance of which has been much discussed (Büttner et al., 2006; Longo et al., 2005; Skulachev, 2002). In terms of kin selection theory, since POD cannot increase personal fitness, to exist it must increase kin fitness which, in turn, implies the presence of social interactions. Investigations of chronological or replicative aging in *S. cerevisiae* aging typically study dispersed cells in shaken culture or micromanipulated individual cells, with little consideration of ecological gerontology (Dytham and Travis, 2006). However, in nature budding yeast exist mainly in spatially-structured, multicellular colonies (Gourlay et al., 2006; Váchová et al., 2012), with distinct patterns of gene expression in different regions of the colony (Wilkinson et al., 2018). PCD has been shown to occur in yeast colonies, mostly in cells in the centre of the colony, and removal of central cells reduces growth of cells at the periphery, suggesting that nutrients released by PCD in the colony core support peripheral cell growth (Váchová et al., 2012; Váchová and Palková, 2005). From one perspective this is an example of POD, where death promotes fitness by kin selection (Büttner et al., 2006), potentially by biomass sacrifice (and perhaps also consumer sacrifice); but from another perspective, it is the colony that is the organism rather than the individual cells within it, in which case PCD here is akin to that in metazoa, i.e. not POD at all (Kirkwood and Melov, 2011).

This applies to PCD in many other "unicellular" species. For example, a variety of bacterial species undergo PCD (Mai-Prochnow et al., 2008; Patton et al., 2005). Again, bacteria often live and grow as multicellular biofilms rather than planktonic suspensions of single cells (Costerton et al., 1999). Consequently there are various potential benefits to PCD in bacterial colonies: not only the transfer of nutrients to kin (biomass sacrifice), but also limiting the spread of viral infection (c.f. *defensive sacrifice*, see below) and protection against DNA damage (Lewis, 2000).

PCD also occurs in unicellular eukaryotes other than *S. cerevisiae*, including chlorophytes and protist parasites (Al-Olayan et al., 2002; Durand et al., 2011; Fabrizio and Longo, 2008). In some cases, these organisms do not form colonies. For example, the trypanosomatid parasite *Leishmania* can undergo PCD (Debrabant et al., 2003), and it is thought that this may dampen the inflammatory response to parasites under attack by host immunity (Zangger et al., 2002). This exemplifies a third type of adaptive death, *defensive sacrifice*, where by dying the altruist protects its relatives from an attacker. Here, in contrast to death after stinging in honey bees (Fig. 2), this is true adaptive death, i.e. death itself provides a benefit. By analogy, the death of a suicide bomber is not beneficial to their cause, but for a captured combatant who commits suicide to avoid betraying their comrades, their death is beneficial.

It is notable that individual *Leishmania* replicate asexually within both sandfly and human hosts, forming clonal populations that exist over extended periods. Thus protected within the host, the relatively low risk of parasitism of the altruist collective by non-related, selfish trypanosomes may permit the evolution of defensive sacrifice. (Thus, according to this scenario, *Leishmania* are parasites to their host, but altruistic to one another).

From this analysis we conclude that in most cases where organisms exhibit PCD and can exist in a unicellular state, PCD probably contributes to fitness in multicellular, colonial forms of those organisms. Here one mechanism by which fitness is promoted appears to be biomass-sacrifice PCD (Váchová et al., 2012). However, defensive sacrifice POD may occur in non-colonial unicells when their niche protects them against selfish parasites (Zangger et al., 2002). Defensive sacrifice can also occur in colonial bacteria (Crespi, 2001; Lewis, 2000), and has also been described in aphids (McAllister and Roitberg, 1987) where it exemplifies altruistic host suicide (Smith Trail, 1980).

In another example of proposed adaptive aging in *S. cerevisiae*, it was recently discovered that during replicative aging there is an increase in variation in capacity to grow on different food sources (Frenk et al., 2017). This suggests that age-changes in budding yeast can increase fitness in the face of a changing nutritional environment, from which the authors draw the thought-provoking conclusion that aging can increase fitness (Frenk and Houseley, 2017; Frenk et al., 2017). Here an easy rationalization is that this represents a maturational change rather than a senescent one. A more interesting possibility suggested by the authors of the study is that senescent (i.e. deteriorative/ degenerative/ pathological) changes lead to the increased substrate range. This draws attention to the absence of a necessary sharp boundary between full biological functionality/full fitness on the one hand and loss of functionality/loss of fitness on the other. This is particularly so given that whether or not a particular biological function promotes fitness or disease can vary according to context (e.g. environmental) (Nesse and Williams, 1994). For example, ROS-induced DNA damage aids adaptive regrowth in *S. cerevisiae* (Fabrizio et al., 2004), which could promote fitness.

We suggest that PCD in organisms that can exist as unicells largely promotes fitness through inclusive fitness and social interactions, via mechanisms such as biomass sacrifice, consumer sacrifice and defensive sacrifice, all consistent with classic evolutionary theory. Biomass sacrifice also supports proliferation of kin during *S. cerevisiae* adaptive regrowth (Fabrizio et al., 2004; Herker et al., 2004). But PCD may also increase fitness by other means, e.g. yeast PCD induced by mating pheromone has been suggested to aid sexual reproduction (Büttner et al., 2006; Severin and Hyman, 2002).

## 7 Adaptive death in *C. elegans*?

Could adaptive death occur in *C. elegans*? Here the type of death we have in mind is not bagging, but that resulting from senescence, as characterised extensively in nematodes cultured (usually) at low density on nutrient agar with a bacterial food source (usually *E. coli*), where their lifespan (20 °C) is 2–3 weeks (Klass, 1977).

### 7.1 Consumer sacrifice in C. elegans?

Based on such laboratory data, consumer sacrifice seems highly unlikely, as the following thought experiment illustrates. Imagine that a single young adult hermaphrodite is placed on an agar plate with a bacterial lawn of unlimited surface area, where it reproduces by selfing, and dies of old age after 18 days. Based on previous estimates of *C. elegans* population growth rate (Venette and Ferris, 1998), by the time the founding hermaphrodite dies, the population of its progeny will have grown to ∼70 billion. Clearly, the benefits of the death of this single parent worm will be vanishingly small, and so selection for consumer sacrifice negligible.

Yet the premises of this thought experiment (e.g. the giant Petri dish etc) are obviously unrealistic. To assess the plausibility of adaptive death, one needs to ask: what are the conditions under which *C. elegans* has evolved? Thanks to work during the last decade, particularly from M.A. Félix, H. Schulenberg et al., much more has recently become known about *C. elegans* ecology. For example, we now know that *C. elegans* are adapted for rapid colonization of transient, patchy (rather than dispersed) food sources, particularly rotting plant stems and fruit (Schulenburg and Félix, 2017). Two features of their life history that particularly affect their existence in the wild are protandrous hermaphroditism, which allows rapid population growth (Hodgkin and Barnes, 1991), and dauer larva formation, enabling long-term survival and dispersal to new food sources (Hu, 2007).

**Fig. 3:**
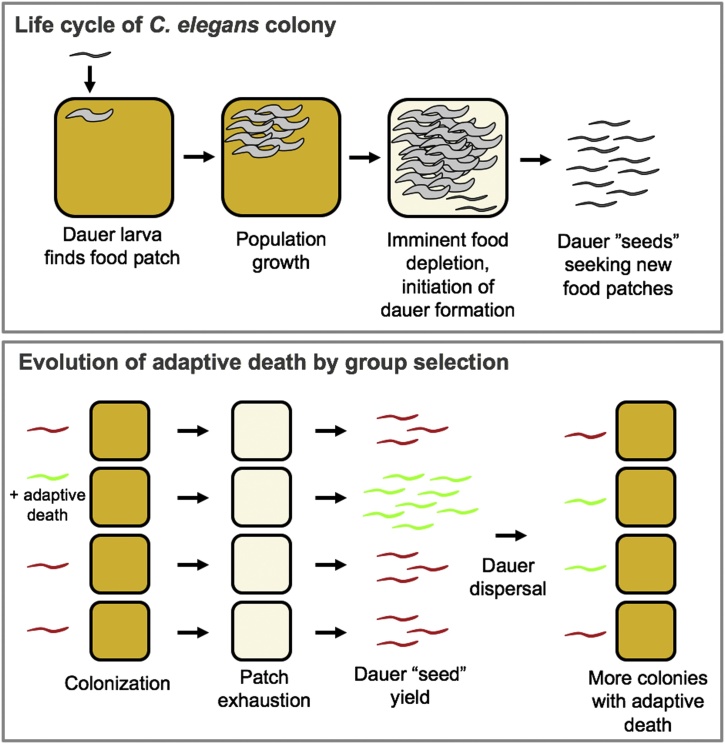
Adaptive death and group selection in *C. elegans* (hypothetical schemes). Top panel, scheme of idealized, simplified life cycle of *C. elegans* colonies. As shown a single dauer larva colonises a food patch, but in reality many dauers and multiple genotypes may colonize the same patch. The colony life cycle ends with production of a yield of dauer larvae, which disperse to new food patches and seed new colonies. Bottom panel, evolution of adaptive death by group selection. Here a hypothetical *C. elegans* ancestor without adaptive death is shown in red, and a new variant with adaptive death is shown in green. In the new variant, adaptive death within the nematode colony increases food availability for population growth, increasing dauer yield. Consequently, adaptive death-positive nematode colonies outcompete their non-altruistic ancestors.

Although little information exists on *C. elegans* lifespan in the wild, it is likely to be far less than that measured in the laboratory. This can be deduced partly from the presence of numerous pathogens and predators found in association with *C. elegans* in the wild, including pathogenic bacteria, fungi, microsporidia and viruses, and predatory fungi and mites (Schulenburg and Félix, 2017). However, given the boom-and-bust existence of *C. elegans* (Frezal and Felix, 2015) the greatest killer is likely to be starvation, since it may be safely deduced that in *C. elegans* populations on rotting plant substrates the largest number of adults will exist just as the food supply begins to run out (Fig. 3, top).

Given *C. elegans*' violent and stressful world, a plausible hypothesis is that senescent changes occurring relative early in adulthood (including those described above) increase adult mortality rate. Supporting this possibility, under laboratory conditions proteostatic collapse reduces heat shock resistance within hours of reproductive maturity (Labbadia and Morimoto, 2015); impairment of proteostasis due to loss of the HSF-1 transcription factor increases susceptibility to a range of bacterial pathogens (Singh and Aballay, 2006); and blocking germline development, which inhibits proteostatic collapse (Labbadia and Morimoto, 2015), also increases resistance to bacterial and fungal pathogens (Alper et al., 2010; TeKippe and Aballay, 2010). Here it is also notable that the fungal pathogen *Drechmeria coniospora* causes NLP-29-dependent acceleration of PVD neurite degeneration in early adulthood (E et al., 2018). If senescence causes significant increases in adult mortality rate soon after reproductive maturity, then consumer sacrifice is no longer ruled out, and the objections from the infinite petri dish scenario may be dismissed.

A second ecological clue about the possibility of adaptive death is that wild *C. elegans* exist largely as populations of selfing hermaphrodites (males are rarely seen), which can be entirely clonal or contain more than one genetically distinct lineage (Barriere and Felix, 2007; Frezal and Felix, 2015; Petersen et al., 2015). The existence of clonal populations in the wild is important because a high level of relatedness in a population increases the possibility of the evolution of altruism through kin selection, as previously noted (Schulenburg and Félix, 2017).

More clues come from computer modelling. Since the beginning of the century, several studies have used computer modelling to explore the possibility of adaptive death, e.g. (Dytham and Travis, 2006; Markov, 2012; Mitteldorf, 2006; Travis, 2004). For example, J.M.J. Travis explored the possibility of adaptive death in a clonal "organism", using a patch occupancy model (Travis, 2004). Here the organisms occupied a grid of 200 × 200 squares. With each time step as the model ran, the organisms could undergo reproduction, programmed death or stochastic (random) death, corresponding to death from extrinsic causes (predation, starvation etc). The model also included an age decrease in reproduction rate. It allowed programmed age of death to evolve in either direction, facilitated by a set mutation rate. The model was then run and tested for the evolution of earlier death (i.e. adaptive/programmed death) under different conditions, e.g. given different rates of age decline in reproduction. The model was run with various modes for offspring dispersal. The dispersal varied from the nearest neighbour mode (to the 8 adjoining patches) to, through increased neighbourhood size, a universal mode where progeny could appear anywhere on the grid (Travis, 2004). As described above, low population dispersal is predicted to allow the evolution of altruism (Fig. 1).

The results of running the model were intriguing in several respects, particularly when viewed in the context of *C. elegans* life history and ecology. First, under some conditions, evolution of earlier programmed death occurred, supporting the possibility of adaptive death. Second, this occurred in the spatially-structured population but not the dispersed population, presumably because in the former case, reproduction was subject to greater constraint by the lack of available squares to be occupied. Given the localized nature of its substrate (rotting plant stems and fruit), proliferating *C. elegans* populations are expected to exhibit low dispersal (Cutter, 2015) conducive to the evolution of altruism.

**Fig. 4:**
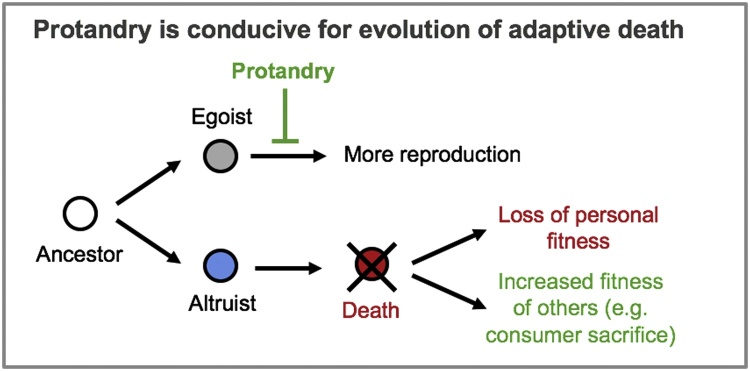
Predicted effects of protandry on the evolution of adaptive death. Alternative means to increasing fitness are living longer and increasing personal fitness by continued reproduction (top), or dying and increasing inclusive fitness through consumer sacrifice (bottom). Protandry (where self-fertilizing organisms produce first sperm and then eggs, such that reproduction ceases upon self-sperm depletion) leads to early cessation of reproduction, thus creating conditions that favor natural selection of altruistic death. *C. elegans* can also reproduce by mating with males, and this extends the reproductive period, but males are very rare in wild populations (Frezal and Felix, 2015). In terms of Hamilton's rule *rB* > *C* where *r* is the relatedness, *B* is the benefit to the recipient, and *C* is the cost to the donor (Hamilton, 1964), protandry results in a smaller reduction in *C* when *B* is increased.

Third, a more rapid age decline in reproduction favored increased programmed death. This is interesting with respect to *C. elegans*, given that it is protandrous, and that a consequence of protandry optimised for maximal population growth rate is early cessation of reproduction (Hodgkin and Barnes, 1991). Thus, Travis' findings are consistent with the view that the evolution of protandry created conditions conducive to the evolution of adaptive death (Fig. 4). This could imply that adaptive death occurs in hermaphroditic species such as *C. elegans* and *C. briggsae*, but not their dioecious sibling species, *C. inopinata* (Kanzaki et al., 2018) and *C. nigoni*. A related model including more biological parameters has confirmed Travis' conclusions (Markov, 2012).

G.C. Williams once wrote: "Intuitions about the evolutionary process can be a great source of ideas but not of conclusions. Conclusions must be based on precise quantitative reasoning, such as realistically formulated mathematical equations or carefully designed graphic models. Such reasoning must be focused in a way that leads to testable expectations about the real world, such as what a series of measurements on a group of fossils will reveal, how an experiment on microorganisms grown in specified environments will turn out, and so on" (Williams, 1996). The outcomes of the Travis model combined with what is now known about *C. elegans* aging and ecology does not prove the occurrence of adaptive death in this organism, but it does suggest that further investigation of this hypothesis is warranted.

Here, it would be helpful to have more information about *C. elegans* ecological gerontology (Dytham and Travis, 2006), particularly life history and population structure in wild (or semi-wild) *C. elegans* populations. It would be useful to know about the real reproductive schedule; for example culture on bacterial species naturally associated with *C. elegans* can lead to delayed reproductive schedule and reduced brood size (Dirksen et al., 2016; Felix and Duveau, 2012); progeny production can also be reduced by infection with microsporidia (Balla et al., 2015; Troemel et al., 2008) and nodaviruses (Ashe et al., 2013). Another parameter affecting reproductive rate is larval mortality rate: what is this under wild conditions? Particularly interesting would be more information about age-specific death rates in adults, and how this changes during the lifetime of *C. elegans* colonies. It would also be interesting to know whether pathological changes in early *C. elegans* adulthood (e.g. proteostatic collapse) increase mortality rate under wild conditions. Also important is the relative frequency on rotting plant substrates of clonal and genetically mixed *C. elegans* populations and, for the latter, their spatial structure; in particular, do older colonies develop a starved core enriched with post-reproductive adults where death benefits the colony (as in *S. cerevisiae*) (Váchová et al., 2012)? Where several genetically distinct *C. elegans* populations share a given substrate, if population viscosity is sufficiently high, then from the point of view of the evolution of altruism, these may be viewed as several distinct populations rather than a single mixed population.

### 7.2 *Biomass sacrifice in* C. elegans*?*

As discussed above, bagging could involve an element of biomass sacrifice. But what about death from senescence in the absence of bagging? This possibility, which is not mutually exclusive with consumer sacrifice, has not been explored experimentally, but several scenarios may be imagined. First, adults dying from senescence may be consumed by other worms in the vicinity; however, the existence of the tough external cuticle might prevent this. Second, dead nematodes could provide a substrate for additional microbial growth, e.g. by species from the intestinal microbiome, which could in turn nourish hungry larvae as populations transition from boom to bust. In either case, resource transfer could involve food-stuffs with caloric value, or rare chemicals such as vitamins (e.g. cholesterol) or, as in salmon, specific elements (e.g. nitrogen, phosphorus).

### 7.3 *Group-level selection in* C. elegans*?*

Group-level selection is plausible in the case of honey bees, where evolution may occur by differential survival and reproduction of individual hives (Seeley, 1997). Could this also be applicable to *C. elegans*? Here it is useful to ask: Do *C. elegans* exist as colonies with superorganism-like features? While *C. elegans* lack complex social behavior such as that holding together a beehive, or physical factors such that those maintaining biofilms of colonial microbes, typical occupation of a spatially-constraining niche (rotting plant stem or fruit) could produce a similar outcome. In the laboratory, *C. elegans* can aggregate in the presence of food and low oxygen tension (Chang et al., 2006), and of indole ascarosides generated by the worms themselves (Srinivasan et al., 2012), and starved L1 larvae show ethanol- and acetate-dependent aggregation (Artyukhin et al., 2015). Moreover, the existence of dauer pheromone as a mechanism for monitoring population density (Golden and Riddle, 1982) is consistent with colony level function (akin to quorum sensing mechanisms in colonial unicells). Low dispersal leads to organism-like spatial structuring of populations which can facilitate evolution of adaptive death that increases inclusive fitness (Dytham and Travis, 2006; Travis, 2004). Thus, *C. elegans* may share features with eusocial organisms.

Viewed as super-organism-like, the life cycle of *C. elegans* could be envisaged as beginning with a dauer larva encountering a rotting plant stem (perhaps by dropping from an isopod vector), and exiting from the dauer state. A clonal population develops, consumes the available food, starves and generates dauer larvae, which disperse (Felix and Duveau, 2012). By this view, much as individuals reproduce by producing eggs, colonies reproduce by producing dauers which disperse to seed new colonies (Fig. 3, top). Here a possibility is that genetic variation between colonies leads to colony-level selection, or at least selection between genetically variant local populations (interdemic selection)(Cutter, 2015; Mitteldorf, 2006; Simon et al., 2013). We postulate that adaptive death can increase colony fitness, manifested as an increase in number of dauer larva colony "seeds", and a consequent increase in number of progeny colonies (Fig. 3, bottom).

In this context it is notable that dauer larvae also associate in a colonial fashion but here caused, as in bees, by behavior. Dauers exhibit a behavior called nictation, where the worms stand on their tails and wave their heads around in the air. This aids in dispersal, allowing dauers to hitch rides e.g. on beetles or isopods, potentially to new feeding grounds. Interestingly, dauers can act cooperatively during nictation, assembling in large numbers to form towers that can reach up to 1 cm in height (Félix and Duveau, 2012); individual dauers are only ∼0.25 mm in length. Notably, the dauers at the base of the dauer towers potentially act altruistically to promote the fitness of those at the top. This is comparable to the colonial behavior of amoeba *Dictyostelium discoideum* (cellular slime mold), which aggregate to form a fruiting body with a head containing spores for dispersal raised up on a stalk. Here the cells forming the stalk cannot reproduce but altruistically enable the cells in the head to reproduce (Wilson and Wilson, 2008). From one perspective, it is not the amoebae that are reproducing here, but the colonial super-organism: the fruiting body.

Whether group selection for adaptive death actually occurs in *C. elegans* will be more easily assessed given better knowledge about its ecological gerontology, including the dynamics of growth and dispersal in wild populations and groups of populations (metapopulations), on which information is currently lacking (Cutter, 2015), though it is clear that genetically distinct *C. elegans* populations can occur on the same food patch (Barriere and Felix, 2007).

Interestingly, the existence of dauer larvae could also promote the evolution of adaptive death. According to kin selection theory, if low dispersal does not alternate with some level of population-wide dispersal, it can hinder the evolution of altruism; this is because like genotypes would then always remain in competition with one another (Bourke, 2011; West et al., 2007). In the case of *C. elegans*, viscous populations of reproducing animals on food patches do indeed alternate with dispersing dauer larvae (Cutter, 2015). Here again linking evolutionary theory to *C. elegans* life history and ecology support the view that adaptive death could have evolved in this organism (we thank A.F.G. Bourke for drawing attention to this). For more on dispersal and adaptive death see (Dytham and Travis, 2006).

## 8 Conclusions, perspectives and speculations

In this essay we argue that results from model organism biogerontology that appear strange in the light of the evolutionary theory of aging are not so strange after all, and that they do not in themselves challenge the classic theory. Instead they reflect the fact that the evolutionary theory is based upon idealized Wright-Fisher populations (i.e. dispersed and out-crossing), a premise from which *C. elegans* and colonial microbes deviate. Other ideas about how aging might promote fitness (e.g. that it promotes evolvability, removes damaged individuals, or assures demographic homeostasis) (Goldsmith, 2008; Mitteldorf, 2006; Skulachev, 2002) have been usefully reviewed elsewhere (Kowald and Kirkwood, 2016).

In most metazoan species (including humans) aging is not an adaptive trait, but where conditions are particularly conducive for the evolution of altruism, e.g. through inclusive fitness, adaptive death can evolve. Permissive conditions include the existence of populations that are viscous and composed of closely-related individuals (Fig. 1). Computer modelling suggests the possibility of adaptive death under such conditions, and that protandry in *C. elegans* could have led to its evolution.

In terms of evolutionary theory, then, the existence of adaptive death in *C. elegans* would not imply anything profoundly novel. By contrast, for *C. elegans* biogerontology, the implications are more interesting. On the positive side, they provide a potential explanation for the existence of mechanisms that dramatically shorten lifespan in *C. elegans*. For example, the proteostatic collapse triggered by reproductive maturity could increase inclusive or colony level fitness by promoting adaptive death. Moreover, these perspectives link *C. elegans* biogerontology to the conceptual riches of evolutionary biology and, we hope, may help to end the fruitless stand-off between these fields - and integration with new knowledge of *C. elegans* ecology, in particular, can facilitate this.

On the negative side, our arguments suggest that insofar as the life-shortening effects of IIS on *C. elegans* function to promote adaptive death, this is a peculiarity of this species, and organisms like it. Although evolutionary conservation of promotion of aging by IIS is renowned (Kenyon, 2010; Partridge and Gems, 2002), the magnitude of effects of IIS on *C. elegans* in terms of proportional changes in lifespan, and effects on aging overall, are far greater than in other organisms examined.

According to the *C. elegans* adaptive death hypothesis *daf-2* may really be the grim reaper after all. Yet its effects on aging cannot be attributable solely to the fitness benefits of adaptive death, for several reasons. First, as just noted, IIS does have some effects on lifespan (albeit smaller) in organisms with Wright-Fisher populations (e.g. *Drosophila*, mouse, probably humans) where adaptive death is not expected to occur. Second, *daf-2* shows some classical AP effects, where pathologies that it promotes are coupled to reproductive fitness (Ezcurra et al., 2018). Third, IIS promotes a wide range of senescent pathologies (Ezcurra et al., 2018; Garigan et al., 2002) which, it may be argued, is an oddly inefficient way to cause organismal death (Blagosklonny, 2013).

### 8.1 A three step hypothesis for the evolution of *C. elegans* adaptive death

To try to explain how IIS might have evolved to promote aging both by AP and adaptive death, we offer an additional, speculative hypothesis. We speculate that *C. elegans* aging evolved via three steps, involving the appearance of (i) protandrous hermaphroditism, (ii) reproductive death, and then (iii) adaptive death, as follows.

In terms of evolution of *C. elegans* aging, a possible role of protandry in the evolution of adaptive death suggests an interesting perspective. Due to the presence of biological constraints, the evolution of a major change in life history can result in some traits that increase fitness but others that reduce it. The latter may lead to secondary adaptations to further increase fitness, a state of affairs that has been likened to the paintings cleverly designed to fit on the surface of the ("non-adaptive") spandrels in the dome at San Marco cathedral in Venice (Gould and Lewontin, 1979).

**Fig. 5:**
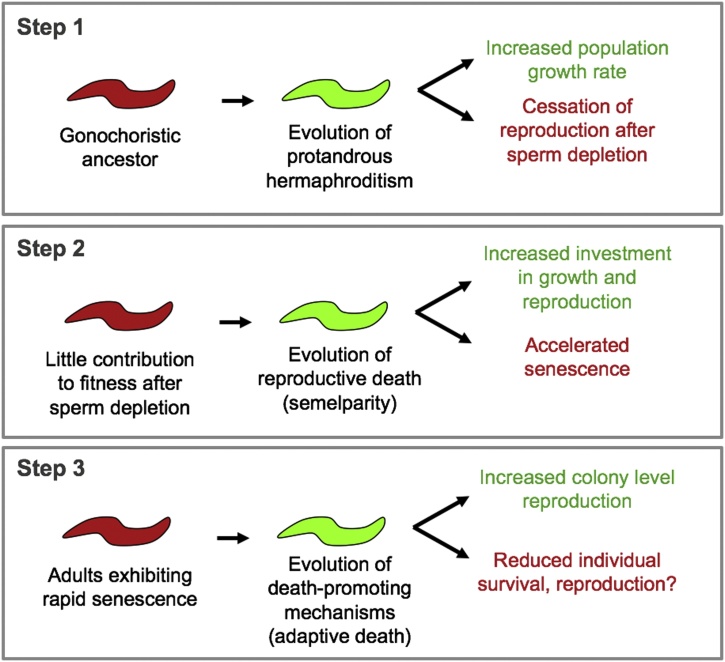
Theory about how adaptive death in *C. elegans* could have evolved by natural selection. Speculative scheme, based on the spandrels of San Marco arguments (Gould and Lewontin, 1979), including a Bauplan-type evolutionary trade-offs model. Here, the evolution of protandry (step 1) increased overall fitness by increasing population growth rate, but reduced some individual fitness elements, particularly by causing cessation of reproduction after sperm depletion (assuming the predominance of selfing in wild populations). Thus, after ∼day 3 of adulthood, animals become futile, and unable to contribution to fitness. This removed evolutionary constraints on deleterious effects of reproduction-senescent pathology trade offs, leading to the evolution of reproductive death: suicidal investment in reproductive effort (step 2). This in turn introduced into populations genetic variation affecting timing of early death with differential effects on colony-level fitness through reproductive death, leading to selection for still earlier death. This model suggests the possible existence of trade offs between individual and colony level fitness. For example, early proteostatic collapse might lead to increased mortality during individual reproduction that reduces individual fitness, e.g. when food availability is high early in colony development, yet increase population growth rate through benefits of consumer sacrifice as food depletion approaches, which will affect a larger number of individual worms.

The evolution of protandrous hermaphroditism in *C. elegans* likely began with the capacity of females to generate and activate self sperm (Baldi et al., 2009), which then triggered a cascade of further evolutionary changes (Ellis and Lin, 2014; Thomas et al., 2012). Hermaphroditism resulted in increased relatedness in nematode populations and an earlier cessation of reproduction due to sperm depletion. We speculate that this may have created conditions in which there evolved first reproductive death (rapid death coupled to major reproductive effort, c.f. Pacific salmon), and then adaptive death (Fig. 5). (The possible occurrence of reproductive death in *C. elegans* will be discussed in a separate essay).

According to this model, in the gonochoristic ancestor of *C. elegans*, IIS coordinated mild trade offs between reproductive fitness and pathology, which were subsequently amplified in the evolution of reproductive death. Consistent with this, there is evidence that DAF-16/FoxO activity is higher in gonochoristic species of *Caenorhabditis* than in *C. elegans* (Amrit et al., 2010). Similarly, down-regulating the UPR might increase reproductive fitness by enabling increased yolk synthesis and secretion (Labbadia and Morimoto, 2014); here it is notable that a decline in proteostasis is also detectable in early adulthood in *Drosophila* (Demontis and Perrimon, 2010; Kawasaki et al., 2016). Subsequently IIS evolved to still higher levels due to fitness benefits conferred by adaptive death, i.e. the late-life AP cost became a benefit (Dytham and Travis, 2006). By this view, the early reproductive redundancy of hermaphrodites that results from protandry corresponds to the spandrels of San Marco to which the pretty painting of reproductive death was added; this led to new spandrels, earlier death, to which the pretty painting of even earlier, adaptive death was then added.

In this essay we have presented the question of existence of programmed, adaptive death in *C. elegans* within a broad context. To this end we have reviewed material from a number of different disciplines (model organism biogerontology, evolutionary biology, ecology), refined and extended terminology and concepts, integrated existing knowledge in new ways to develop several novel hypotheses and speculative models. Our hope is that the ideas presented stimulate and usefully inform future studies into the evolutionary biology of aging in *C. elegans*, particularly its ecological gerontology (Dytham and Travis, 2006).

## Conflict of interests

The authors declare that there is no conflict of interests regarding the publication of this manuscript.

## Glossary

**Gonochoristic**: Where adults are male or female (as opposed, in the context of this essay, to male or hermaphrodite)
**Protandry**: The form of gametogenesis in hermaphroditic organisms where male gametes are formed first and then female gametes. *C. elegans* hermaphrodites generate sperm and then oocytes. Sperm are used for self-fertilization, and when self sperm stocks are depleted, reproduction ceases
**Programmed aging**: Senescence caused by a relatively ordered series of biological processes that promotes fitness via inclusive fitness or group fitness
**Quasi-programmed aging**: Senescence caused by a relatively ordered series of biological processes that does not promote fitness; may occur due to futile run-on of wild-type programs that promote fitness earlier in life (Blagosklonny, 2006)
**Fitness (direct Darwinian fitness)**: The contribution of the reproductive success of an individual with a specified genotype to the gene pool of the next generation
**Inclusive fitness**: Sum of an individual’s direct Darwinian fitness and its indirect fitness derived from the reproductive success of descendants and close relatives
**Kin selection**: Evolutionary strategy that increases the fitness of an individual’s relatives, even at a cost to the individual’s own Darwinian fitness, which can occur if the individual’s inclusive fitness increases as a consequence
**Viscous population**: A population where individuals closely related by ancestry remain in close physical proximity to one another, as in colonial populations; the opposite of a dispersed population (Fig. 1)
**Group selection**: Natural selection between entire groups of organisms. Occurs where an evolved trait provides a benefit to one group over another
**Multilevel selection** (MLS): The idea that selection acts not only on individuals but simultaneously on multiple levels of biological organization. Traits that benefit other individuals are selectively disadvantageous at the level of the individual, but they might still evolve if they are advantageous at - and hence selected for on - a higher level of the biological hierarchy (e.g. on the group or colony level)(Fig. 2, top)
**MLS-1 MLS-2**: MLS-1 group fitness is defined as the average (or total) fitness of its constituent organisms (Fig. 1, bottom), whereas MLS-2 group fitness is defined as the expected number of offspring groups (Fig. 3, bottom)
**Ecological gerontology**: Combining information about ecology and aging to understand how the ecology of an organism shapes the evolution of senescence, and how senescence (and traits to which it is coupled) impacts population ecology (Dytham and Travis, 2006)
**Adaptive death**: Synonymous with programmed organismal death. Here death of an individual is a selected trait, providing a direct benefit in terms of inclusive or group fitness
**False adaptive death** (New term): Where a trait or behavior that promotes fitness results in immediate death, but where death does not itself increase fitness (Fig. 2, bottom). This is merely a form of benefit-cost trade off, where the cost occurs immediately after the benefit (e.g. matriphagy)
**Consumer sacrifice** (New term): A mechanism by which adaptive death promotes fitness, where by dying an individual increases food availability for kin, thereby increasing their reproduction and hence increasing its inclusive fitness, or by increasing group fitness (Fig. 2, top)
**Biomass sacrifice** (New term): A mechanism by which adaptive death promotes fitness, where death enhances transfer of an individual's own biomass to its kin, thereby increasing reproduction of kin and hence its inclusive fitness, or by increasing group fitness (Fig. 2, top)
**Defensive sacrifice** (New term): A mechanism by which adaptive death promotes fitness, where by dying an individual protects its kin from attack, e.g. by predators, host immunity or pathogens; c.f. altruistic host suicide (Smith Trail, 1980) (Fig. 2, top)
**Reproductive death**: A form of suicidal reproductive effort found in some semelparous species (e.g. Pacific salmon, monocarpic plants). Here, reproductive maturity triggers the rapid development of lethal pathologies and fast senescence coupled to reproductive success (Finch, 1990)
